# Acyl-CoA-binding protein (ACBP): the elusive ‘hunger factor' linking autophagy to food intake

**DOI:** 10.15698/cst2019.10.200

**Published:** 2019-09-24

**Authors:** José Manuel Bravo-San Pedro, Valentina Sica, Frank Madeo, Guido Kroemer

**Affiliations:** 1 INSERM U1138, Centre de Recherche des Cordeliers, Sorbonne Université, Université de Paris, 15 rue de l'école de médecine 75006, Paris, France.; 2 Team “Metabolism, Cancer & Immunity” labellisée par la Ligue contre le Cancer, Paris, France.; 3 Cell Biology and Metabolomics platforms, Gustave Roussy Cancer Campus, Villejuif, France.; 4 Institute of Molecular Biosciences, NAWI Graz, University of Graz, Graz, 8010, Austria.; 5 BioTechMed Graz, Graz, 8010, Austria.; 6 Pôle de Biologie, Hôpital Européen Georges Pompidou, AP-HP, Paris, France.; 7 Suzhou Institute for Systems Medicine, Chinese Academy of Sciences, Suzhou, China.; 8 Karolinska Institute, Department of Women's and Children's Health, Karolinska University Hospital, Stockholm, Sweden.

**Keywords:** appetite control, diazepam binding protein, metabolism, obesity, unconventional protein secretion

## Abstract

The best-known appetite-regulating factors identified in rodents are leptin, an appetite inhibitor, and ghrelin, an appetite stimulator. Rare cases of loss-of-functions mutations affecting leptin and its receptor, as well as polymorphisms concerning ghrelin and its receptor, have been documented in human obesity, apparently validating the relevance of leptin and ghrelin for human physiology. Paradoxically, however, the overwhelming majority of obese individuals manifest high leptin and low ghrelin plasma levels, suggesting that both factors are not directly disease-relevant. We recently discovered that acyl-CoA-binding protein (ACBP), also known as diazepam-binding inhibitor (DBI), acts as an efficient lipogenic and appetite stimulator in mice. Indeed, in response to starvation, ACBP/DBI is released from tissues in an autophagy-dependent fashion and increases in the plasma. Intravenous injection of ACBP/DBI stimulates feeding behavior through a reduction of circulating glucose levels, and consequent activation of orexigenic neurons in the hypothalamus. In contrast, neutralization of ACBP/DBI abolishes the hyperphagia observed after starvation of mice. Of note, ACBP/DBI is increased in the plasma of obese persons and mice, pointing to a convergence (rather than divergence) between its role in appetite stimulation and human obesity. Based on our results, we postulate a novel ‘hunger reflex' in which starvation induces a surge in extracellular ACBP/DBI, which in turn stimulates feeding behavior. Thus, ACBP/DBI might be the elusive ‘hunger factor' that explains increased food uptake in obesity.

Overweight has beaten undernutrition as the most frequent pathological state throughout the world, affecting close to 25% of the adult population. This has severe implications for global health, given that obesity is the major risk factor for most if not all non-communicable diseases, including the entire spectrum of cardiovascular, neoplastic, metabolic and neurodegenerative diseases. Among the G20 countries, the US is the uncontested leader (adult obesity rate ~36%) followed by countries with a rate of 30-35% (Saudi Arabia, Turkey), a large group of countries with a rate of 20-30% (Argentina, Australia, Brazil, Canada, France, Germany, Mexico, Russia, South Africa, United Kingdom), one European Country that is just undercutting 20% (Italy) and a group of Asian Countries with obesity rates well under 10% (China, India, Indonesia, Japan, South Korea). These numbers (http://worldpopulationreview.com) eloquently underscore the cause of the obesity pandemic, which is the Western lifestyle characterized by excessive consumption of calories (and in particular carbohydrates and ultra-processed food) coupled to sedentarism, as well as the failure of public health education [1–4].

In spite of the extremely high prevalence of obesity, multiple studies have been designed to define genetically determined ‘risk factors' that would explain why only one third of the population reaches a body mass index (BMI) >30 [5–7]. Such studies were spurred by the discovery of leptin, the satiety hormone. Loss-of-function mutation of leptin in mice causes hyperphagy and obesity (in *Ob*/*Ob* mice), as does that of its receptor (in *Db*/*Db* mice) [8, 9]. Later exceptionally rare cases of human obesity with mutations in the genes coding for leptin or its receptor were described [10, 11]. However, the vast majority of obese patients exhibit an increase in circulating leptin levels (perhaps as a failing homeostatic mechanism in which leptin levels are upregulated yet fail to tame appetite), meaning that leptin deficiency is not a major pathogenic factor in obesity [12–14]. Other studies have led to the discovery of a major appetite-stimulatory factor, ghrelin, in rodents [15]. However, obese patients exhibit a decrease in circulating ghrelin levels (again, likely as a failing homeostatic mechanism), indicating that excessive ghrelin cannot be the cause of human obesity [16, 17]. Moreover, in patients with anorexia nervosa, ghrelin levels are paradoxically high [18, 19], while leptin levels are paradoxically low [20, 21]. These results indicate that major appetite control circuitries discovered in mice cannot be pharmacologically manipulated to prevent or treat human eating disorders.

In eukaryotes, autophagy is the phylogenetically most ancient response to dwindling nutrient resources, allowing cells and organisms to sequester and to digest non-essential macromolecules contained in the cytoplasm [22, 23]. Continuous or periodic stimulation of autophagy by caloric restriction or intermittent fasting, respectively, improves the fitness of model organisms ranging from yeast to primates [24–28]. Indeed, autophagy is the mechanisms through which constant or periodic limitations in food access increases the healthspan and lifespan of model organisms [29–31]. Caloric excess suppresses autophagy, thereby abolishing an important cytoplasmic recycling mechanism, favoring the storage of excessive lipid in a variety of cell types, reducing cellular and organismal fitness, and likely precipitating the manifestation of age-related diseases, which are the ‘co-morbidities' of obesity [32, 33]. Indeed, obesity is linked to a state of autophagic suppression [34] and autophagy induction by pharmacologic manipulations has anti-obesity effects [35], suggesting that autophagy inhibition is causally involved in the pathogenic cascade that leads to supraphysiological adiposity [33, 36].

Intrigued by these insights, we have been attempting to develop ‘caloric restriction mimetics' (CRMs), i.e. pharmacological agents that mimic the biochemical effects of caloric restriction [37–39]. Nutrient deprivation causes autophagy induction through the depletion of the cytosolic pool of acetyl coenzyme A (AcCoA), resulting in deacetylation of cytoplasmic proteins (including a number of proteins involved in the regulation and execution of autophagy), thereby stimulating autophagic flux [40, 41]. CRMs mimic the effect of caloric restriction because they inhibit enzymes that generate AcCoA (such as ATP citrate lyase) or that use AcCoA for protein acetylation (such as the EP300 acetyltransferase) or, alternatively, stimulate deacetylases (such as sirtuin 1), resulting in autophagy induction [38, 39]. The collection of CRMs includes several compounds reputed for their capacity to extend healthspan and/or lifespan such as aspirin [42], chalcones [43], resveratrol [44] and spermidine [30, 45]. This latter agent is a natural polyamine present in food items. Epidemiological studies suggest that ingestion of high levels of spermidine reduces overall mortality as well as disease-specific mortality from cancer and cardiovascular disorders [46–48], supporting prior evidence in yeast, nematodes, fruit flies and mice that spermidine delays age-associated disease and death [30, 45].

Given that autophagy seems to antagonize obesity-associated disease pathogenesis [35, 49], we searched for novel ways to stimulate this process. Back in 2010, several groups reported that fungal species can release one particular protein, acyl-coenzyme A binding protein (ACBP, also known as diazepam-binding inhibitor, DBI) in an autophagy-dependent fashion [50–52]. Based on the fact that cell stress is usually communicated to other cells, a phenomenon that can be referred to ‘inside-outside communication' [53], we wondered whether extracellular ACBP/DBI protein might be a target for modulating autophagy or even impact on pathogenic processes. In mice and humans, ACBP/DBI is ubiquitously expressed (though particularly high in adipocytes, https://www.proteinatlas.org/). As indicated by its dual name, ACBP/DBI has two functions, as an intracellular buffer and transporter for acyl coenzyme A, and as a modulator of benzodiazepine receptors (and in particular the gamma-aminobutyric acid (GABA) A receptor) [54–56]. Intriguingly, we found that any type of human or murine cell released ACBP/DBI upon starvation (which is the most physiological stimulus of autophagy) *in vitro* and *in vivo* through a process that can be inhibited by deletion of essential autophagy genes or by pharmacological autophagy inhibitors [57]. Thus, the autophagy-associated release of intracellular ACBP/DBI into the extracellular space appears to be a general, phylogenetically conserved phenomenon that applies to both fungal and mammalian systems.

We then set out to determine the effects of ACBP/DBI on autophagy and general metabolism. Interestingly, in human and mouse cell cultures the depletion of intracellular and extracellular ACBP/DBI do not have the same effects on autophagy. Depletion of intracellular ACBP/DBI by small interfering RNAs (siRNAs) inhibits autophagy, while neutralization of extracellular ACBP/DBI with suitable antibodies stimulates autophagy [57]. These results may be interpreted to mean that the autophagy-associated secretion of ACBP/DBI is involved in a negative feedback loop limiting autophagy. In mice, fasting was associated with an increase in the plasma concentration of ACBP/DBI, and intravenous injection of recombinant ACBP/DBI protein inhibited starvation-induced autophagy, while ACBP/DBI neutralization (by means of an intraperitoneally injected antibody) enhanced autophagy [57].

Intravenous injection of recombinant ACBP/DBI protein had multiple effects on metabolism (**Fig. 1**) including a rapid (30 min) increase in the expression of the glucose transporter GLUT1 on hepatocytes. This was accompanied by a reduction in plasma glucose levels that could be prevented by GLUT1 inhibitors. Experiments involving isotope-labelled glucose revealed the presence of labeled-glucose in the adipose tissue a few hours after ACBP/DBI injection. ACBP/DBI concomitantly inhibited fatty acid oxidation. Most importantly, mice injected with ACBP/DBI manifested a close-to-immediate (30 min) hyperphagic response that was accompanied by the activation of orexigenic neurons in the hypothalamus. When glucose levels were maintained in an artificial fashion (by injection of glucose into the peritoneal cavity) both hyperphagy and the activation of orexigenic neurons were prevented, suggesting that the effects of ACBP/DBI on central appetite control were secondary to its metabolic effects on peripheral tissues. Of note, in this time frame ACBP/DBI injection did not affect insulin or ghrelin levels. Of note, sustained overexpression of a transgene coding for ACBP/DBI in hepatocytes was sufficient to cause a significant increase in weight gain coupled to an augmentation of perigonadal and visceral adiposity [57].

**Figure 1:**
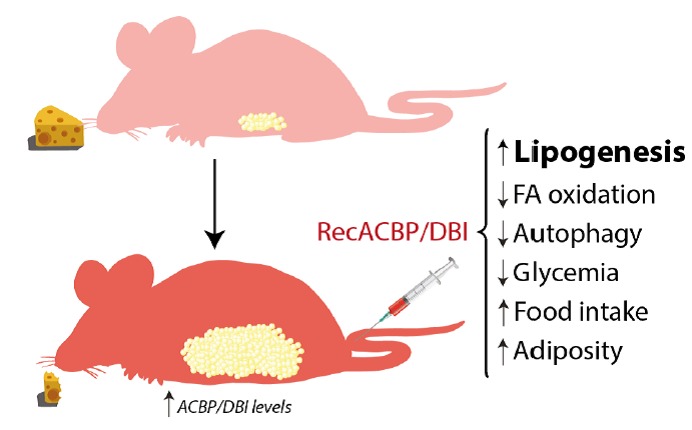
FIGURE 1: Effects of recombinant ACBP/DBI in mice. Metabolic effects detailed after intravenous injection of recombinant ACBP/DBI (recACBP/DBI) protein, including an increase of lipogenesis, food intake and consequent adiposity and decrease of autophagy, glycemia and fatty acid (FA) oxidation.

Altogether, the aforementioned data suggest that ACBP/DBI is an orexigenic and obesogenic factor. In accord with this interpretation, neutralization of ACBP/DBI had anorexigenic and lipolytic effects (**Fig. 2**). Thus, the hyperphagic response of mice that had been starved for 24 hours (which causes ~10% weight loss) was largely abolished by intraperitoneal injection of neutralizing ACBP/DBI antibodies, which, in parallel, prevented the reduction of glycemia that normally accompanies a 24-hour fasting period, caused a decrease in circulating insulin levels, and caused the activation of anorexigenic neurons in the hypothalamus. In mice, ACBP/DBI neutralization led to increased lipolysis from white adipose tissue, an increase in gluconeogenesis from glycerol (which may explain the maintenance of glucose levels), as well as an important raise in fatty acid oxidation. ACBP/DBI neutralization could be achieved for longer periods by a specific immunization protocol designed to break autotolerance and to elicit autoantibodies against ACBP/DBI. The surge in neutralizing ACBP/DBI autoantibodies led to a reduction in weight gain induced by high-fat diet (in normal mice) or by feeding a normal diet to leptin-deficient (*Ob/Ob*) mice. These effects were accompanied by a reduction in the abundance of white adipose tissue, a reduction in the median diameter of adipocytes, browning of fat, amelioration of the glucose tolerance test, as well as a reduction of hepatosteatosis. Of note, an inducible whole-body knockout of ACBP/DBI recapitulated many of these features, suggesting that the predominant effect of both (intracellular + extracellular) pools of ACBP/DBI is indeed orexigenic and obesogenic [57]. In a plausible scenario, ACBP/DBI would be involved in a ‘hunger reflex' in which starvation leads to a transient, autophagy-dependent release of ACBP/DBI from tissues, and extracellular ACBP/DBI then causes metabolic changes that ultimately stimulate feeding behavior, favor lipo-anabolic reactions and inhibit catabolic pathways including autophagy (**Fig. 3**).

**Figure 2:**
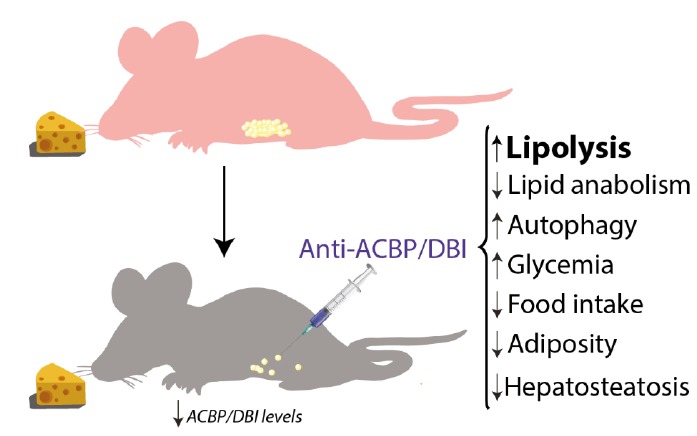
FIGURE 2: ACBP/DBI neutralization and its effects in mice. Metabolic effects observed after intraperitoneal injection of neutralizing anti-ACBP/DBI antibody, including an increase of lipolysis, glycemia and autophagy and decrease of lipogenesis, food intake, hepatosteatosis and body weight.

**Figure 3:**
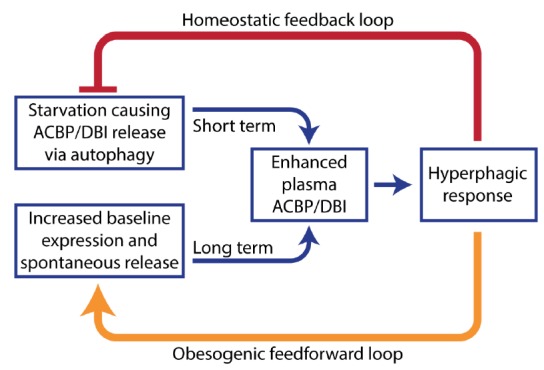
FIGURE 3: The ‘hunger reflex'. Hypothetical explanation of the pathogenesis of obesity. Extracellular ACBP/DBI participates in a short-term homeostatic feedback loop to link starvation to food intake. However, obesity causes an increase in ACBP/DBI expression and its spontaneous release that leads to chronic hyperphagy, thus locking the disease in a pathogenic feedforward loop.

We also examined the levels of ACBP/DBI expression in patients with anorexia and obesity. Of note, we found a high (Spearman r>0.8) positive correlation between the body mass index and plasma ACBP/DBI levels across several patient cohorts. Thus, anorexic patients manifested subnormal ACBP/DBI plasma concentrations, while obese individuals were characterized by supranormal ACBP/DBI. After successful bariatric surgery ACBP/DBI levels decline when patients lose weight, but increase again when they relapse. Dietary interventions that cause transient weight loss also temporarily reduce *ACBP/DBI* mRNA expression in the periumbilical fat tissue [57]. In mice, we observed a similar trend. Murine obesity was associated with higher ACBP/DBI plasma concentrations, as well as with increased *ACBP/DBI* mRNA and protein expression in the liver and white adipose tissue. In obese humans, we found a positive association between, on one hand, plasma ACBP/DBI and, on the other hand, fasting insulin levels as well as aspartate transaminases (AST). Thus, ACBP/DBI correlates with laboratory parameters indicative of insulin-resistant (type 2) diabetes and liver damage [57]. However, such clinical observations do not allow to establish any cause-effect relationships beyond these correlations.

Altogether, these results support the notion that ACBP/DBI has not only an obesogenic function in mice but that it is indeed increased in obesity in humans. Thus, at difference with leptin and ghrelin, ACBP/DBI exhibits a concordant (rather than discordant) behavior in mice and in humans with eating disorders (**Table 1**). At this stage, we postulate that ACBP/DBI may well be the elusive ‘hunger factor' that is elevated in obesity. Obviously, clinical studies must be designed to neutralize ACBP/DBI or its receptor and to validate this assumption.

**Table 1.** Comparison among major appetite control systems in mice and human obesity.

	**Mouse**	**Human**	**Observation**
Leptin	Genetic deficiency of leptin or its receptor causes hyperphagy and obesity.	High in obesity Low in anorexia nervosa	**Paradoxical** association of human hyperphagy with high levels of an anorexigenic factor.
Ghrelin	Administration of ghrelin causes hy-perphagy and obesity.	Low in obesity High in anorexia nervosa	**Paradoxical** association of human hyperphagy with low levels of an orexigenic factor.
ACBP/DBI	ACBP/DBI administration causes hy-perphagy and obesity, while neutrali-zation of ACBP/DBI is anorexigenic.	High in obesity Low in anorexia nervosa	**Concordant** association of human hyperphagy with high levels of an orexigenic factor.

## Abbreviations:

ACBP: – Acyl-CoA-binding protein,
AcCoA: – acetyl coenzyme A,
CRM: – caloric restriction mimetic,
DBI: – diazepam-binding protein.
